# Treatment condition as a moderator and change in trait mindfulness as a mediator of a brief mindfulness ecological momentary intervention for generalized anxiety disorder

**DOI:** 10.1192/j.eurpsy.2024.1750

**Published:** 2024-05-07

**Authors:** Nur Hani Zainal, Michelle G. Newman

**Affiliations:** 1 Department of Health Care Policy, Harvard Medical School, Boston, MA, USA; 2 Department of Psychology, National University of Singapore, Kent Ridge, Singapore; 3 Department of Psychology, The Pennsylvania State University, University Park, PA, USA

**Keywords:** causal inference, change mechanism, ecological momentary intervention, generalized anxiety disorder, mediation, mindfulness, randomized controlled trial

## Abstract

**Background:**

Theories propose that judgment of and reactivity to inner experiences are mediators of the effect of mindfulness-based interventions on generalized anxiety disorder (GAD). However, no study has tested such theories using brief, mindfulness ecological momentary intervention (MEMI). We thus tested these theories using a 14-day MEMI versus self-monitoring app (SM) control for GAD.

**Methods:**

Participants (*N* = 110) completed self-reports of trait mindfulness (Five Facet Mindfulness Questionnaire), GAD severity (GAD-Questionnaire-IV), and trait perseverative cognitions (Perseverative Cognitions Questionnaire) at prerandomization, posttreatment, and 1-month follow-up (1MFU). Counterfactual mediation analyses with temporal precedence were conducted.

**Results:**

Improvement in pre–post mindfulness domains (acceptance of emotions, describing feelings accurately, acting with awareness, judgment of inner experience, and reactivity to inner experience) predicted pre-1MFU reduction in GAD severity and pre-1MFU reduction in trait perseverative cognitions from MEMI but not SM. MEMI reduced pre–post reactivity to inner experiences (but not other mindfulness domains) significantly more than SM. Only reduced pre–post reactivity significantly mediated stronger efficacy of MEMI over SM on pre-1MFU reductions in GAD severity (indirect effect: β = −2.970 [−5.034, −0.904], *p* = .008; *b* path: β = −3.313 [−6.350, −0.276], *p* = .033; percentage mediated: 30.5%) and trait perseverative cognitions (indirect effect: β = −0.153 [−0.254, −0.044], *p* = .008; *b* path: β = −0.145 [−0.260, −0.030], *p* = .014; percentage mediated: 42.7%). Other trait mindfulness domains were non-significant mediators.

**Conclusions:**

Reactivity to inner experience might be a mindfulness-based intervention change mechanism and should be targeted to optimize brief MEMIs for GAD.

## Introduction

Mindfulness-based interventions (MBIs) aim to improve attention focused on the present moment, purposefulness, and non-judgmental awareness [1]. Meta-analytic data from randomized controlled trials (RCTs) indicated that both therapist-led [2] and entirely self-guided [3, 4] MBIs were significantly effective in mitigating anxiety, depression, and associated common mental health symptoms. Nevertheless, there remains uncertainty regarding whether MBI outcomes can be unequivocally attributed to a particular change mechanism. Understanding the mechanisms behind the effectiveness of MBIs might assist clinicians and policymakers in pinpointing the therapeutic targets to prioritize, thus potentially enhancing efficacy by initiating essential change processes [5]. Consequently, it is imperative to conduct MBI trials to evaluate potential change mechanisms.

MBIs are believed to operate by focusing non-judgmental attention on the present moment and enhancing disciplined attention toward a task. They teach people to persistently cultivate these skills in the face of challenges [6]. Due to the focus of MBIs on the present moment, disciplined mindfulness exercises counteract psychopathological symptoms, such as excessive worry about potential future threat, which is central to generalized anxiety disorder (GAD) [7]. Overall, these theories posit that trait mindfulness would serve as a mediator of the impact of MBIs on mental health outcomes.

Five trials offered consistent evidence for this mediation hypothesis. Three single-arm trials showed that increased global trait mindfulness mediated the impact of MBIs on reduction in GAD severity [8] and perceived stress [9-11]; however, the absence of a control group precluded ruling out regression to the mean and expectancy effects and limited internal validity and causal inferences. A two-arm waitlist-controlled RCT showed that increased pre–post global trait mindfulness mediated the effect of MBSR on lower posttreatment distress and avoidance in cancer patients [12]. Despite that, this RCT had only two assessment waves and thus could not specify temporal precedence in a change-to-change causal chain as recommended [13]. In a three-arm RCT that exemplified best mediation practices, increased pre-mid global trait mindfulness mediated the effect of a fully self-guided internet-delivered MBI against waitlist and active control on reducing pre–post stress among college students [14]. However, focusing on global trait mindfulness limits understanding of how *specific* domains might act as mediators in understanding MBI change mechanisms. Improving our comprehension of which specific trait mindfulness domains act as stronger mediators than others in enhancing outcomes can facilitate the precise customization of MBIs.

Factor analyses have classified trait mindfulness domains into five categories [15, 16]. *Observing* pertains to paying attention to or recognizing inner and outer experiences, that is, auditory input, feelings, olfactory sensations, thoughts, and visual cues. *Describing* entails mentally recognizing or labeling experiences using language*. Acting with awareness* refers to focusing on present actions instead of engaging in autopilot or inattentive behavior. *Judgment of inner experience* is the tendency to form negative opinions about one’s feelings, sensations, and thoughts, for example, berating oneself for feeling upset after a breakup rather than processing emotions such as sadness without judgment. *Reactivity to inner experience* indicates a resistant and non-accepting response to one’s fleeting feelings and thoughts instead of letting feelings naturally come and go. An example of reactivity includes resisting feelings of doubt while working on a project instead of accepting the feeling and allowing it to pass naturally, thereby adversely affecting focus on the task. Higher judgment and reactivity to inner experience tended to coincide with lower trait mindfulness and more frequent repetitive thinking [17, 18].

To maximize the benefits of MBIs in reducing GAD symptoms and related perseverative cognitions, it may be crucial to specifically enhance two distinct trait mindfulness domains: decreased judgment and reduced reactivity to inner experiences. This proposition is based on consistent evidence that GAD was marked by excessive reactivity and inflexible beliefs about the “utility” of worry to protect oneself from sharp increases in negative emotions rather than mindfully allowing emotions to ebb and flow [19, 20]. Subjectively, heightened GAD severity has been uniquely correlated with higher judgment and reactivity [21]. Further, individuals with (versus without) GAD self-reported heightened emotional intensity and more difficulty bouncing back from strong increases in negative emotions [22, 23]. They also experienced an increased sense of threat and reduced emotional control [24-26]. Interpersonally, persons with (versus without) GAD were more reactive to the negative emotions of others during social interactions [27]. Neurologically, they exhibited increased amygdala sensitivity when expecting an adverse event [28]. Physiologically, people with (versus without) GAD showed delayed autonomic recovery when confronted with emotionally charged situations [29]. The *contrast avoidance model* proposes that persons with GAD fail to practice mindful non-reactivity to inner experiences and instead use worry to increase and sustain negative emotions to avoid intense reactivity to stressors or abrupt spikes in negative emotions [19, 30]. There is also a tendency in GAD toward negatively biased interpretations about ambiguous issues [cf. cognitive model; 31, 32]. Thus, refraining from judgment is essential. According to these theories and evidence, MBIs may need to reduce reactivity and judgment to effectively decrease worry and other repetitive thoughts in these individuals.

Despite these theories, no trials have tested how changes in specific trait mindfulness domains preceded and mediated reductions in symptoms and if treatment group moderated such mediation effects in the context of GAD. However, six trials have examined how distinct trait mindfulness domains might mediate the effect of MBIs against controls on other mental health outcomes. For example, pre–post increased observing and reduced reactivity to inner experience mediated the effect of an MBI against waitlist on pre–post reduction in depression symptoms in stressed meditation-naïve individuals [33]. However, its non-randomized and two-time-point design permitted only correlational inferences. In addition, four RCTs that reported how reduced reactivity [34, 35], judgment [36], and enhanced acting with awareness [37] mediated the effect of MBI against waitlist or treatment-as-usual on clinical outcomes in non-psychiatric samples failed to examine treatment arm as a moderator. An RCT that reported how increased non-reactivity to inner experience mediated the effect of mindfulness ecological momentary intervention (MEMI) versus treatment-as-usual on pre-follow-up worry also did not test treatment as a moderator [38]. Relatedly, an exemplary moderated mediation analysis using RCT data showed that acting with awareness mediated the effect of MEMI versus waitlist on distress among non-depressed school employees [39]; despite that, this study only examined one trait mindfulness domain as a mediator. Also, a qualitative review proposed that decreases in judgment and reactivity might be necessary for MBIs to alleviate symptoms of anxiety disorders, including worry [40]. Together, the diverse mediating effects with distinct clinical endpoints highlight the importance of testing *unique* trait mindfulness domains to uncover potential change mechanisms underlying MBIs for GAD.

This study thus determined what specific trait mindfulness domain(s) might mediate the effect of a 14-day MEMI against a self-monitoring app (SM) on GAD severity and trait perseverative cognitions. Previously, we showed the efficacy of MEMI against SM in reducing GAD severity and trait perseverative cognitions at pre-1-month follow-up (pre-1MFU) [4]. Our present study aimed to improve on prior studies in four ways. First, we ensured optimal temporal sequence such that random assignment preceded pre–post change in the mediator, and pre–post change in the mediator preceded pre-1MFU change in outcome. Only two of the 11 prior trials implemented this recommendation [14, 36]. Second, we built on previous research by testing how the results were generalizable to a clinical sample of people diagnosed with GAD. Third, most prior studies tested 4–16-week in-person MBIs, and none tested how trait mindfulness domain(s) might have been a change mechanism of *brief, fully self-guided* MEMIs. Brief MBIs have been defined as those lasting up to 2 weeks [41]. This aim was essential as people with GAD have tended to face stigma, shame, time, and travel constraints to seeking treatment and would instead prefer to solve problems independently [42], necessitating thorough evaluation of digital, fully self-guided MEMIs. Fourth, we tested if pre–post change in trait mindfulness domains was a mediator and assigned intervention was a moderator, based on recommendations [43]. Based on theory and evidence, we examined the hypotheses that MEMI would yield efficacy over SM by reducing pre–post judgment of and reactivity to inner experience (versus the other three domains) in reducing pre-1MFU GAD severity (Hypothesis 1) and trait perseverative cognitions (Hypothesis 2).

## Method

### Participants

We enrolled 110 participants who met the study inclusion criteria, with 68 randomized to MEMI and 42 to SM. They were drawn from both the local community and psychology subject pool. Table 1 presents the demographic and clinical attributes of the participants. Also, there were no significant between-group variations in the occurrence of concurrent psychiatric diagnoses at baseline.

**Table 1.** Sociodemographic data of study participants in the MEMI and SM (*N* = 110)

	MEMI (*n* = 68)		SM (*n* = 42)		*p*
Continuous variables	*M*	(SD)	*M*	(SD)	
Age (in years)	20.53	(3.91)	21.24	(7.24)	.51
14–item GADQ–IV score	9.52	(2.10)	9.94	(1.96)	.30
Treatment expectations					
Credibility	6.00	(1.39)	5.72	(1.58)	.34
Expectancy	43.46	(17.33)	44.29	(18.13)	.31
Categorical variables	*n*	(%)	*n*	(%)	*p*
Gender orientation					.85
Men	10	(14.71)	5	(11.90)	
Women	57	(83.82)	37	(88.10)	
Declined to disclose	1	(1.47)	–	–	
Race					.99
White Caucasian	44	(64.71)	27	(64.29)	
Asian or Asian American	11	(16.18)	4	(9.52)	
Hispanic	3	(4.41)	5	(11.91)	
African American	5	(7.35)	1	(2.38)	
Another race	4	(5.88)	2	(4.76)	
Declined to disclose	1	(1.47)	0	(0.00)	
Comorbid diagnoses					
Current major depressive episode	32	(47.10)	24	(57.10)	.30
Recurrent major depressive episode	25	(36.80)	20	(47.60)	.26
Current panic disorder	16	(23.50)	5	(11.90)	.13
Current social anxiety disorder	15	(22.10)	14	(33.30)	.19
Current OCD	4	(5.88)	4	(9.52)	.48
Current PTSD	9	(13.20)	4	(9.52)	.56
Current alcohol use disorder	7	(10.30)	1	(2.38)	.12
Current substance use disorder	3	(4.41)	1	(2.38)	.58
Current anorexia nervosa	0	(0.00)	0	(0.00)	–
Current binge–eating disorder	1	(1.47)	0	(0.00)	.39

Abbreviations: MEMI, mindfulness ecological momentary intervention; OCD, obsessive-compulsive disorder; PTSD, post-traumatic stress disorder; SM, self-monitoring app.

### Study design and eligibility criteria

Our RCT (registered under NCT04846777 on ClinicalTrials.gov, with the mediation analyses preregistered on Open Science Framework: https://osf.io/63jcr) obtained ethical clearance from a state university in the eastern United States. It utilized a mixed-design approach involving two intervention groups (MEMI and SM) assessed at three time points (prerandomization, postintervention, and 1MFU). Time served as the within-participant variable, whereas group functioned as the between-participant variable.

Participants meeting the diagnostic criteria for GAD according to the Diagnostic and Statistical Manual-Fifth Edition (DSM-5) [44] were eligible for inclusion in the study. They were also required to be treatment-seeking and not currently in mental health treatment. Additionally, participants needed to be ≥18 years of age, possess a smartphone running either the iOS or Android operating system, and provide informed consent. Initial screening included the Generalized Anxiety Disorder Questionnaire-Fourth Version [GADQ-IV; 45] and the following questions, “Are you currently receiving any treatment for psychological difficulties?” and “Are you currently interested in seeking treatment for psychological difficulties?” The GADQ-IV includes both binary (“Yes” or “No” questions) and continuous response options, such as a 9-point Likert scale, to measure the impact and distress caused by GAD symptoms. It aligns with the DSM-5 GAD criteria [44]. Those whose GAD-Q-IV scores met or exceeded the clinical cutoff [46] received the Anxiety and Related Disorders Interview Schedule for DSM-5 [ADIS-5; 47] to confirm their mental health diagnoses. It was delivered by trained and supervised research assistants in-person (prepandemic) or over Zoom (during the pandemic). Exclusion criteria were the presence of suicidal ideation, manic episodes, psychotic disorders, or substance use disorders, assessed by the ADIS-5.

### Intervention groups

**MEMI.** All MEMI participants received an informative video featuring the lead investigator, a clinical psychologist with a PhD. This video conveyed essential elements of evidence-based MBI protocols, aligning with the principles found in MBSR [1]. MEMI participants were provided clear instructions on mindfulness, encouraging them to engage fully in their present surroundings, current activity, or task at hand. This section was designed to help individuals who are chronically worried to develop the skill of open monitoring, improving their ability to focus on small details. Next, the video therapist guided participants on intentional, rhythmic, and slowed diaphragmatic breathing techniques, followed by a practical demonstration of the correct execution. This component offered guidance on practices promoting serenity through controlled breathing exercises and cultivating mindful attributes such as non-reactive observation and non-judgment, inspired by the principles of MBCT [48]. Later, the video therapist stressed the importance of integrating mindfulness into daily routines. Participants received a MEMI rationale document delivered automatically through Qualtrics to maintain the evaluator-blinding design. The document specifically directed them to review and engage in mindfulness exercises.

MEMI prompted individuals to engage in mindfulness activities at five specific times during each day: approximately 9 a.m., noon, 3 p.m., 6 p.m., and 9 p.m., spanning 14 days. During each MEMI prompt, participants received standard directives: “Pay attention to your breathing. Breathe in a slow, steady, and rhythmic manner. Stay focused on sensations of the air coming into your lungs and then letting it out. As you are breathing, observe your experience as it is. Let go of judgments that do not serve you. Focus on the here and now. Attend to the small moments right now (e.g., reading a chapter, having a cool glass of water), as that is where enjoyment, peace, and serenity in life happen.” Before and after each prompt, participants rated their present levels of mindfulness (“To what extent are you experiencing the present moment fully?”), depression, and anxiety (“To what degree do you feel depressed/[keyed up or on edge] right now?”) on a 9-point scale (1 = *Not At All* to 9 = *Extremely*). Each MEMI alert concluded with a message to encourage the long-term integration of these skills: “Remember that the cultivation of mindfulness is lifelong. The goal of therapy is to be your own therapist. Practice mindfulness between the prompts and after you have completed this study.”

**SM.** In SM, the standardized video began with the therapist explaining self-monitoring as heightened awareness of one’s emotional states and thought processes. Afterward, the video proposed to individuals engaging in self-monitoring that carefully observing their thoughts and recording any linked emotional discomfort might help them develop beneficial cognitive-emotional processes. Ultimately, the SM video conveyed the idea that the practice of self-observation alone might alleviate anxious feelings. The fundamental basis for the SM control condition was drawn and modified from the rationale used in a recent brief app intervention [49, 50]. This strategy was crafted to closely mirror the MEMI protocol but excluded its presumed beneficial elements, such as acceptance, being present, diaphragmatic breathing, and continual mindfulness exercises. As a result, it purposely avoided any reference to the mindfulness concepts and refrained from explicitly instructing participants to heighten their awareness and perception of their present experiences. Instead, it emphasized observing their distressing emotional reactions and thoughts at each prompt. At the same time, we omitted instructions for accepting these thoughts and feelings as they arose. SM participants were also not directed to focus solely on their current tasks. In addition, these individuals did not receive instructions on breathing retraining methods to induce pleasant sensations associated with relaxation. Also, they were not encouraged to continue self-observation beyond the designated prompts or after the initial 14-day intervention phase ended. The aim of the SM was to minimize credibility and expectancy effects, prevent regression to the mean, and avoid potential overestimation of effect sizes commonly observed in no-treatment/waitlist control groups [51].

Unlike the detailed mindfulness guidance provided by MEMI, SM participants received a brief single-sentence instruction five times daily (around 9 a.m., 12 p.m., 3 p.m., 6 p.m., and 9 p.m.) for 14 days: “Notice your thoughts and how distressing they may be.” We assessed participants’ mindfulness, depression, and anxiety levels using identical 9-point Likert scale questions before and after each prompt during every SM signal. Participants were also provided with an automated copy of the SM handout. Unlike MEMI, this handout did not include instructions to review its contents regularly.

### Measures

**Trait mindfulness domains.** Trait mindfulness was assessed using the Five Facet Mindfulness Questionnaire (FFMQ), a self-report tool consisting of 39 items aimed at measuring mindfulness practices in everyday life [15, 16]. As mentioned earlier, it included five trait mindfulness domains: *observing* (eight items; e.g., “I pay attention to how my emotions affect my thoughts and behavior.”), *describing* (e.g., “I can usually describe how I feel at the moment in considerable detail.”), *acting with awareness* (e.g., “I find myself doing things without paying attention.”), *judgment of inner experience* (e.g., “I disapprove of myself when I have irrational ideas.”), and *reactivity to inner experience* (e.g., “When I have distressing thoughts or images, I just notice them and let them go.”). The FFMQ subscale scores have shown strong convergent and discriminant validity [52], effectively distinguishing themselves from measures of unrelated constructs such as psychological well-being [16]. FFMQ subscale scores have also shown high retest reliability [53]. Participants rated items on a 5-point scale (1 = *never or very rarely true* to 5 = *very often or always true*). Our internal consistency (Cronbach α) values were high at prerandomization, posttreatment, and 1MFU, respectively, for the observing domain (αs = .75, .87, .92) and other subscales (describing: .92, .86, .91; acting with awareness: .86, .88, .92; judgment of inner experience: .90, .89, .93; reactivity to inner experience: .82, .85, .90).

**GAD severity.** GAD severity was assessed using the 16-item GAD-Q-dimensional measure, which resembles the 14-item GADQ-IV but consistently features response formats on a 9-point Likert scale (e.g., 0 = *never* to 8 = *almost every day*, 0 = *not at all* to 8 = *worry all the time*). The first eight questions of the GADQ-Dimensional focused on evaluating enduring worry traits. Respondents rated the extent, frequency, manageability, and strength of their worries. The following eight questions asked about similar worries during the past 6 months (possible score range = 0–126; αs = .90, .92, .93).

**Trait perseverative cognitions.** The Perseverative Cognitions Questionnaire (PCQ), consisting of 45 items, assessed persistent, trait-level repetitive negative thinking patterns associated with obsessions, worry, and rumination [54]. Participants indicated their agreement with items on a 6-point Likert scale (0 = *strongly disagree* to 5 = *strongly agree*). Moreover, the PCQ comprised six distinct factors: lack of controllability, preparing for the future, expecting the worst, searching for causes/meanings, dwelling on the past, and thoughts discordant with ideal self. The overall PCQ score was derived by summing the average scores of each subscale. The PCQ has demonstrated robust convergent validity, discriminant validity, 2-week retest reliability [54], and cross-cultural measurement equivalence [55]. Our internal consistency values were also high (possible score range = 0–6; αs = .96, .97, .97).

### Procedures

During the initial visit, participants underwent the structured ADIS-5 interview. Eligible participants then completed a series of self-reports, cognitive functioning, and social cognition assessments before randomization. This process was counterbalanced to mitigate any potential biases related to the order of assessments. The evaluators remained unaware of the assigned groups by physically leaving the room (pre-COVID-19 pandemic) or by instructing participants to turn off their Zoom audio and video before opening the Qualtrics link to watch the assigned group video (peri-pandemic). Participants downloaded the PACO app (https://github.com/google/paco), preloaded with MEMI or SM, onto their smartphones following a video tutorial. The evaluator was available to address any inquiries participants had about study procedures, such as upcoming study visits or technical issues related to installing PACO on their phones. However, the evaluator was absent during participants’ introduction to their assigned intervention arm and its components. After a 14-day intervention phase, all participants returned for posttreatment assessments and then again at the 1-month follow-up (1MFU), 6 weeks from baseline. During these sessions, they completed standardized self-reports and other assessments. Participants received compensation in the form of credit hours, monetary payment, or a combination of both. On the seventh day, evaluators conducted a compliance check to examine if participants completed at least 56/70 prompts as instructed.

### Data analyses

Missing data, which accounted for 10.71% of the total dataset, were addressed using random forest imputation with the *missRanger R* package [56]. To test the efficacy of MEMI against SM on domain-specific trait mindfulness mediator targets, we utilized an intent-to-treat methodology similar to the approach used in the primary efficacy analysis [4]. This method utilized a multilevel model, where changes in outcome over time were determined by differences from pre-1MFU, with group as the between-participant factor. For multilevel mediation analysis, we used a causal mediation model called the marginal mediation model [57]. Traditional mediation models (e.g. [58]) presuppose that unmeasured factors do not affect the mediator-outcome associations, an assumption known as “sequential ignorability” [59]. Since we defined the pre–post mediator as change in potential targets (observing, describing, acting with awareness, judgment of inner experience, reactivity to inner experience) preceding the pre-1MFU outcome, participants were not randomly assigned to the different mediator levels [60]. The marginal mediation model diverges from the sequential ignorability assumption by establishing a connection between mediation parameters and causal parameters [60]. The marginal mediation model evaluated the significance of three multiplicative paths: MEMI versus SM predicting pre-1MFU outcome (*c* path or direct effect), MEMI versus SM predicting potential pre–post mediator (*a* path), and pre–post potential mediator predicting pre-1MFU outcome (*b* path). Controlling for random assignment simultaneously, this analysis represented the pure indirect effect [60]. Temporal precedence was established following best practices, ensuring that random assignment preceded the pre–post mediator and the pre–post mediator preceded the pre-1MFU outcome [61]. Simple slope analyses were conducted to examine within-group parameter estimates. Each potential mediator was analyzed individually. Given the theoretical significance of each mediator and their intercorrelations, we refrained from controlling for other mediators [62]. We displayed the unstandardized regression coefficients (β) with 95% confidence intervals (CIs) and utilized bootstrapping with 1,000 resampling iterations [63]. Sensitivity analyses were performed using non-linear generalized additive multilevel models to assess the consistency of the observed findings [64]. The Simes alpha correction method was utilized [65]. The effect size was calculated as the ratio of the indirect effect to the total effect [66]. Three *R* packages – *intmed* [67], *mediation* [64], and *mgcv* [68] – were used with adapted tutorials from published sources (e.g., http://tinyurl.com/codesintmed; http://tinyurl.com/codesmediation).

## Results

### Intervention effect on pre–post trait mindfulness mediators (path a)

**Figure 1.**
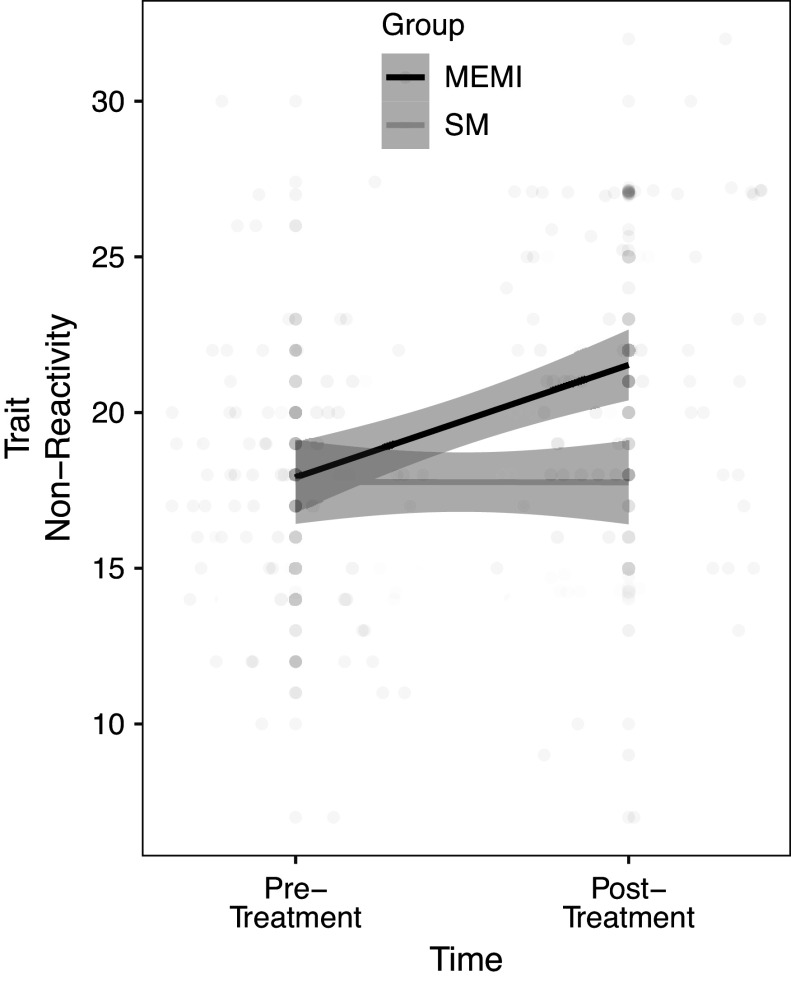
Efficacy of MEMI versus SM on pre–post trait nonreactivity to inner experience. MEMI, mindfulness ecological momentary intervention; SM, self-monitoring app.

MEMI was significantly more effective than SM in reducing pre–post reactivity to inner experience (β = 1.578 [0.525, 2.631], *p* = .003) but not observing (β = 1.264 [−0.091, 2.619], *p* = .067), describing (β = 0.795 [−0.496, 2.086], *p* = .227), acting with awareness (β = 1.039 [−0.281, 2.359], *p* = .123), and judgment (β = −0.404 [−1.927, 1.119], *p* = .602; Figure 1). Simple slope analyses indicated that MEMI significantly improved reactivity (β = 1.806 [0.987, 2.625], *p* < .001), unlike SM (β = −0.007 [−0.955, 0.941], *p* = .988). Although MEMI did not induce pre–post changes in other mediators to a greater degree than SM, MEMI significantly enhanced pre–post observing (β = 1.262 [0.154, 2.370], *p* = .026), describing (β = 0.997 [0.077, 1.916], *p* = .034), acting with awareness (β = 1.441 [0.434, 2.448], *p* = .005) and reduced judgment (β = 2.274 [1.099, 3.449], *p* < .001) (Tables 2 and 3). SM did not significantly change pre–post observing (β = 0.121 [−0.999, 1.241], *p* = .831), describing (β = 0.579 [−0.790, 1.949], *p* = .404), acting with awareness (β = 0.260 [−1.003, 1.522], *p* = .685), and judgment (β = 0.734 [−0.690, 2.157], *p* = .310).

**Table 2.** Simple slope analyses of predictor-mediator and mediator-outcome associations for pre-1MFU GAD severity as the outcome

	Predicting the pre–post mediator (*a* path)			Predicting pre-1MFU GAD severity (*b* path)		
	β	(LCI, UCI)	*p*	β	(LCI, UCI)	*p*
A. Observing						
MEMI	1.262*	(0.154, 2.370)	.026	−5.770^***^	(−9.029, −2.511)	.000
SM	0.121	(−0.999, 1.241)	.831	−1.071	(−5.267, 3.126)	.615
B. Describing						
MEMI	0.997*	(0.077, 1.916)	.034	−6.230*	(−9.560, −2.900)	< .001
SM	0.579	(−0.790, 1.949)	.404	−0.489	(−4.519, 3.541)	.811
C. Acting with awareness						
MEMI	1.441^**^	(0.434, 2.448)	.005	−4.928^***^	(−8.069, −1.786)	.002
SM	0.260	(−1.003, 1.522)	.685	−0.691	(−4.580, 3.198)	.726
D. Judgment (reverse–coded)						
MEMI	2.274^***^	(1.099, 3.449)	.000	−4.612^***^	(−7.863, −1.360)	.006
SM	0.734	(−0.690, 2.157)	.310	−0.358	(−4.386, 3.669)	.861
E. Reactivity to inner experience (reverse–coded)						
MEMI	1.806^***^	(0.987, 2.625)	.000	−3.423^***^	(−6.528, −0.319)	.031
SM	−0.007	(−0.955, 0.941)	.988	−1.040^***^	(−4.805, 2.724)	.585

*Note:* * *p* < .05; ^**^ *p* < .01; ^***^ *p* < .001.

Abbreviations: 1MFU, 1-month follow-up; β, unstandardized regression coefficient; GAD, generalized anxiety disorder; LCI, lower bound of the 95% confidence interval (CI); MEMI, mindfulness ecological momentary intervention; SM, self-monitoring app; UCI, upper bound of the 95% CI.

**Table 3.** Simple slope analyses of predictor-mediator and mediator-outcome associations for pre-1MFU trait perseverative cognitions as the outcome

	Predicting the pre–post mediator (*a* path)			Predicting pre-1MFU trait perseverative cognitions (*b* path)		
	β	(LCI, UCI)	*p*	β	(LCI, UCI)	*p*
A. Observing						
MEMI	1.262*	(0.154, 2.370)	.026	−0.253^***^	(−0.380, −0.126)	.000
SM	0.121	(−0.999, 1.241)	.831	−0.077	(−0.245, 0.092)	.370
B. Describing						
MEMI	0.997*	(0.077, 1.916)	.034	−0.279^***^	(−0.413, −0.145)	.000
SM	0.579	(−0.790, 1.949)	.404	−0.049	(−0.206, 0.108)	.539
C. Acting with awareness						
MEMI	1.441^**^	(0.434, 2.448)	.005	−0.218^***^	(−0.339, −0.097)	.000
SM	0.260	(−0 1.003, 1.522)	.685	−0.068	(−0.232, 0.097)	.418
D. Judgment (reverse–coded)						
MEMI	2.274^***^	(1.099, 3.449)	.000	−0.180^***^	(−0.302, −0.058)	.004
SM	0.734	(−0.690, 2.157)	.310	−0.044	(−0.203, 0.114)	.580
E. Reactivity to inner experience (reverse–coded)						
MEMI	1.806^***^	(0.987, 2.625)	.000	−0.133^***^	(−0.246, −0.019)	.023
SM	−0.007	(−0.955, 0.941)	.988	−0.076	(−0.226, 0.074)	.318

*Note:* * *p* < .05; ^**^ *p* < .01; ^***^ *p* < .001.

Abbreviations: 1MFU, 1-month follow-up; β, unstandardized regression coefficient; GAD, generalized anxiety disorder; LCI, lower bound of the 95% confidence interval (CI); MEMI, mindfulness ecological momentary intervention; SM, self-monitoring app; UCI, upper bound of the 95% CI.

### Pre–post trait mindfulness mediator predicting pre-1MFU GAD severity (path b)

Treatment significantly moderated the pathways of all pre–post trait mindfulness domains predicting pre-1MFU change in GAD severity: observing (β = −6.155 [−9.452, −2.858], *p* < .001), describing (β = −6.019 [−9.268, −2.771], *p* < .001), acting with awareness (β = −4.893 [−7.981, −1.804], *p* = .002), judgment (β = −4.614 [−7.809, −1.419], *p* = .005), and reactivity (β = −3.313 [−6.350, −0.276], *p* = .033). Within the MEMI, larger increase in pre–post observing (β = −5.770 [−9.029, −2.511], *p* < .001), describing (β = −6.230 [−9.560, −2.900], *p* < .001), acting with awareness (β = −4.928 [−8.069, −1.786], *p* = .002), and decreased judgment (β = −4.612 [−7.863, −1.360], *p* = .006), and reactivity (β = −3.423 [−6.528, −0.319], *p* = .031) significantly predicted greater reduction in pre-1MFU GAD severity (Table 2). However, within the SM, changes in pre–post observing (β = −1.071 [−5.267, 3.126], *p* = .615), describing (β = −0.489 [−4.519, 3.541], *p* = .811), acting with awareness (β = −0.691 [−4.580, 3.198], *p* = .726), judgment (β = −0.691 [−4.580, 3.198], *p* = .726), and reactivity (β = −1.040 [−4.805, 2.724], *p* = .585) did not significantly predict change in pre-1MFU GAD severity.

### Pre–post trait mindfulness mediator predicting pre-1MFU trait perseverative cognitions (path b)

Treatment significantly moderated the pathways of all pre–post trait mindfulness domains predicting pre-1MFU change in perseverative cognitions: observing (β = −0.274 [−0.406, −0.143], *p* < .001), describing (β = −0.276 [−0.405, −0.146], *p* < .001), acting with awareness (β = −0.239 [−0.364, −0.114], *p* < .001), judgment (β = −0.194 [−0.317, −0.072], *p* = .002), and reactivity (β = −0.145 [−0.260, −0.030], *p* = .014). Within the MEMI, larger increase in pre–post observing (β = −0.253 [−0.380, −0.126], *p* < .001), describing (β = −0.279 [−0.413, −0.145], *p* < .001), acting with awareness (β = −0.218 [−0.339, −0.097], *p* < .001), and decreased judgment (β = −0.180 [−0.302, −0.058], *p* = .004), and reactivity (β = −0.133 [−0.246, −0.019], *p* = .023) significantly predicted greater reduction in pre-1MFU perseverative cognitions. However, within the SM, changes in pre–post observing (β = −0.077 [−0.245, 0.092], *p* = .370), describing (β = −0.049 [−0.206, 0.108], *p* = .539), acting with awareness (β = −0.068 [−0.232, 0.097], *p* = .418), judgment (β = −0.044 [−0.203, 0.114], *p* = .580), and reactivity (β = −0.076 [−0.226, 0.074], *p* = .318) did not significantly predict change in pre-1MFU perseverative cognitions.

### Intervention effect on pre-1MFU GAD severity via pre–post trait mindfulness domains (indirect effect)

In the total sample, reduction in pre–post reactivity to inner experience significantly mediated the effect of MEMI against SM predicting a larger decrease in pre-1MFU GAD severity (β = −2.970 [−5.034, −0.904], *p* = .008; effect size: 30.5%). However, pre–post change in observing (β = −0.566 [−1.488, 0.040], *p* = .074), describing (β = −0.543 [−1.601, 0.407], *p* = .226), acting with awareness (β = −1.286 [−3.039, 0.328], *p* = .140), and judgment (β = 0.346 [−1.158, 1.804], *p* = .618) were not significant mediators of MEMI against SM on pre-1MFU GAD severity. Effect sizes were small (3.9–13.4%) for these non-significant mediation paths. A sensitivity analysis that examined non-linear mediator-outcome relations using multilevel generalized additive models led to similar findings (Table S1 in the online supplemental materials). Hypothesis 1 thus received partial support.

### Intervention effect on pre-1MFU trait perseverative cognitions via pre–post trait mindfulness domains (indirect effect)

In the total sample, stronger reduction in pre–post reactivity to inner experience significantly mediated the effect of MEMI against SM predicting greater decrease in pre-1MFU perseverative cognitions (indirect effect: β = −0.153 [−0.254, −0.044], *p* = .008; effect size: 42.7%). However, pre–post change in observing (β = −0.043 [−0.099, 0.002], *p* = .064), describing (β = −0.033 [−0.093, 0.020], *p* = .224), acting with awareness (β = −0.057 [−0.134, 0.014], *p* = .100), and judgment (β = 0.022 [−0.055, 0.110], *p* = .598) were not significant mediators of MEMI against SM predicting pre-1MFU perseverative cognitions. Effect sizes were small (6.3–16.2%) for these non-significant mediation paths. A sensitivity analysis that examined non-linear mediator-outcome relationships produced similar findings (Table S2 in the online supplemental materials). Hypothesis 2 was, therefore, partially supported.

## Discussion

Partially affirming our hypotheses, pre–post reduction in reactivity to inner experience emerged as a crucial moderated mediator – potentially a change mechanism – of the effect of MEMI against SM on pre-1MFU reductions in GAD severity and trait perseverative cognitions. Stated differently, decrease in reactivity to inner experiences accounted for 30.5–42.7% of the effect of brief MEMI against SM in mitigating pathological worry and other patterns of repetitive negative thinking. Pre–post change in other trait mindfulness domains – observing, describing, acting with awareness, and judgment of inner experience – did not serve as mediators for the intervention effect on clinical outcomes. Our outcomes indicated that other mediators apart from reactivity to inner experiences were not proxy change mechanisms of brief MEMI in treating GAD [69]. At the same time, change in all mindfulness domains predicted subsequent changes in pathological worry and GAD severity. Theoretical accounts are provided to elucidate these findings, potentially offering valuable insights for future research endeavors exploring similar moderated mediational analyses in RCTs of MBIs for GAD or related conditions.

What potential change mechanisms might explain the efficacy of MEMI on reactivity to inner experiences? Behaviorally, the MEMI might have helped chronic worriers discern their emotions, then pause, observe, and respond wisely while staying present instead of reacting negatively to internal feelings, thoughts, or sensations better than SM [70, 71]. Cognitively, the MEMI might have done a better job than SM at helping to decrease reactivity to rumination and worry [72, 73]. Biologically, the MEMI, as with other MBIs, could have attenuated the cortisol awakening response [a marker of stress reactivity; 74, 75-77]. Future digitally delivered MBI RCTs that include multimodal measures could test the validity of these ideas.

Why did the pre–post decrease in reactivity to inner experience emerge as the only mediator of treatment effect on reducing GAD severity and trait perseverative cognitions at pre-1MFU? Maybe MEMI bolstered resilience to stressors [78]. In light of this, our findings can be contextualized by evidence indicating that individuals with GAD tended to exhibit heightened reactivity [19]. Physiologically, prolonged worry has been causally linked to decreased vagal tone [i.e., higher resting heart rate; 24] and increased blood pressure [79]. Neurobiologically, people with versus without GAD showed hyperactivity in the amygdala when seeing unpleasant pictures [80]. The inclination toward pathobiological reactivity in GAD may be partially attributed to brain-derived neurotrophic factors and related genetic factors [81].

Other behavioral and cognitive factors might also explain why reduction in reactivity to inner experience mediated the effect of MEMI against SM on decreases in GAD severity and trait perseverative cognitions at pre-1MFU. Behaviorally, people with GAD self-rated higher levels of intensity in their emotional experiences than depressed people [82]. Further, worry consistently amplified and prolonged negative emotional states and thus increased the likelihood of feeling less negative in the absence of dreaded events or feeling more positive in the presence of positive ones [19, 30]. These patterns consistently manifested in daily life across different situations, with worry initiating and maintaining anxiety while predicting a decreased likelihood of significant increases in negative emotions in future periods [22, 23, 83, 84]. Cognitively, GAD has been associated with increased focus on threats [85], the tendency to interpret ambiguous material negatively [86], and executive dysfunction [87]. In summary, targeting reduction in reactivity to inner experience could enhance the effectiveness of brief MEMIs for GAD by honing specific skills to mitigate emotional or stress reactivity across multiple biopsychosocial dimensions.

Despite recent theories proposing that reduced judgment of inner experience could be a crucial trait mindfulness domain mediator explaining treatment effects of MBIs for anxiety disorders [40], our findings did not align with those assertions. However, it is important to note that in MEMI (but not SM) pre–post reduced judgment (and improvement in all other mindfulness domains) did predict pre-follow-up reductions in both trait perseverative cognitions and GAD severity. Therefore, reduced judgment was associated with pre-follow-up outcomes even though it was not a mediator. It may not have been a differential mediator because there was no between-treatment effect on judgment from pre-to-posttreatment [88]. It is possible that enhancing the intensity of MEMI over longer periods was needed for reduced judgment to act as a moderated mediator [89]. More intense treatment might raise the odds of finding a differential reduction in judgment in MEMI (versus SM) and of reduction in judgment as a differential mediator perhaps because learning to simply observe without immediately forming opinions of experiences as “good” or “bad” may be an attitude that takes time to cultivate [90].

Interestingly, although there were no significant between-group differences, it is worth noting that within-group analyses of change revealed that MEMI, unlike SM, improved pre–post observing, describing, and acting with awareness, while also reducing judgment and reactivity. These findings might be explained by evidence suggesting that MBIs, compared to active controls, were more effective in enhancing state and trait attentional skills [91], executive functioning [92], and emotional clarity [93]. Encouragingly, prior research has shown improvement in all these mindfulness domains following an 8-week MBSR course compared to a waitlist in healthy controls [52], suggesting that similar benefits might extend to 14-day MEMIs for individuals with GAD. In addition, pre–post enhancements in all trait mindfulness domains predicted reductions in GAD severity and perseverative cognitions at pre-1MFU in MEMI but not SM. MEMI may have been more effective than SM in teaching the skill of observing experiences without an immediate reaction, improving emotion regulation with more constructive responses and fewer detrimental coping strategies [94]. Further, evidence that MBIs better equipped people with GAD and depression with the skills to perceive emotions and thoughts as transient occurrences rather than personally associating with them – a process called “decentering” – than controls [8, 95] might explain our findings.

This study had a number of limitations. First, although temporal precedence was established, it is essential to note that mediation alone does not necessarily provide a complete understanding of the underlying change mechanism [69]. Further evidence of causality through experiments establishing mediator-outcome relations would be essential, coupled with coherent theories explaining the mechanism(s) by which causation operates in the process [96]. Secondly, the short intervention phase may not have allowed sufficient time for significant differential pre–post improvements in all trait mindfulness domains, except for reactivity to inner experience. Further, our study did not include assessments of the continued utilization of mindfulness skills by MEMI participants from postintervention to the 1MFU. Future RCTs testing digitally delivered MBIs should investigate whether sustained mindfulness engagement, even without repeated MEMI instructions, could influence treatment effects during assessments from postintervention to follow-up. Also, the conclusions drawn from our study may not apply to a broader demographic beyond predominantly White female participants. This limitation underscores the importance of future digital trials attracting a more diverse participant pool, encompassing various cultural backgrounds, genders, and related diversity metrics.

However, the current study had notable strengths, including its utilization of an RCT design with an active control group and a high level of participant engagement. Further, we recruited a clinical sample through face-to-face diagnostic assessment and included one month follow-up assessments. Our study also had a dropout rate of only 11%, which was significantly lower than the typical range of 24–50% observed in mental health RCTs delivered via smartphones [97-99]. Another strength was the rigor of our causal mediation modeling approach, which extended traditional approaches [61].

If our observed results are replicated, several clinical implications merit consideration. As decreases in reactivity to inner experience emerged as the sole noteworthy mediator, this finding suggests that clients with GAD should not resist sharp increases in negative emotional states by sustained dampening of their emotions. Instead, they should accept and embrace all kinds of transient emotions that arise in their field of experience. Such an approach might alleviate worry and other perseverative cognitions, thereby optimizing the effectiveness of brief MEMI for GAD [100]. Further, guiding clients with GAD on managing distressing thoughts and emotions without impulsive reactions could be beneficial. Regularly practicing reduced reactivity to emotionally challenging situations could help maintain focus on mood-boosting activities, thereby reducing worrisome and unhelpful thinking patterns [101]. Further, clinical psychological science can benefit from identifying individuals for whom reactivity to inner experience and other trait mindfulness domains might act as proxy mechanisms of change in brief, cost-effective, self-guided MEMIs, enhancing their dissemination within stepped-care and stratified care frameworks [102, 103].

## Supporting information

Zainal and Newman supplementary material Zainal and Newman supplementary material

## Supplementary material

The supplementary material for this article can be found at http://doi.org/10.1192/j.eurpsy.2024.1750.
