# Supplementary material for: Treatment condition as a moderator and change in trait mindfulness as a mediator of a brief mindfulness ecological momentary intervention for generalized anxiety disorder
Source: Eur Psychiatry. 2024 May 7;67(1):e40. doi: 10.1192/j.eurpsy.2024.1750 (PMC11363003; doi:10.1192/j.eurpsy.2024.1750)
Supplement: Zainal and Newman supplementary material [file S0924933824017504sup001.docx]

**Online Supplemental Materials**

Table S1

*Causal mediation analyses of pre-post trait mindfulness domains mediating the effect of MEMI vs. SM on pre-1MFU GAD severity using non-linear generalized additive models*

|  | Predicting the pre-post mediator  (*a* path) | | |  | Predicting pre-1MFU GAD severity  (*b* path) | | |
| --- | --- | --- | --- | --- | --- | --- | --- |
|  | β | (LCI, UCI) | *p* |  | β | (LCI, UCI) | *p* |
| A. Observing | | | |  |  | | |
| Group x Time | 1.264 | (-0.086, 2.614) | .067 |  | -7.843^***^ | (-12.169, -3.516) | .000 |
| B. Describing | | | |  |  | | |
| Time | 0.837^*^ | (0.072, 1.603) | .033 |  | -4.091^***^ | (-6.592, -1.59) | .001 |
| C. Acting with awareness | | | |  |  | | |
| Group x Time | 1.039 | (-0.276, 2.354) | .123 |  | -8.410^***^ | (-12.458, -4.363) | .000 |
| D. Non-judgment | | | |  |  | | |
| Group x Time | -0.404 | (-1.921, 1.114) | .602 |  | -10.112^***^ | (-14.243, -5.981) | .000 |
| E. Non-reactivity to inner experience | | |  |  |  | | |
| Group x Time | 1.578^**^ | (0.529, 2.627) | .003 |  | -6.729^***^ | (-10.663, -2.795) | .001 |
| Spline regressions | | | |  | *F* | *p* |  |
| A. Observing | | | |  | 10.088^***^ | .000 |  |
| B. Describing | | | |  | 16.267^***^ | .000 |  |
| C. Acting with awareness | | | |  | 56.352^***^ | .000 |  |
| D. Non-judgment | | | |  | 45.850^***^ | .000 |  |
| E. Non-reactivity to inner experience | | | |  | 86.483^***^ | .000 |  |

*Note.* ^*^ *p* < .05; ^**^ *p* < .01; ^***^ *p* < .001.

MEMI, mindfulness ecological momentary intervention; SM, self-monitoring app; 1MFU, one-month follow-up; β, unstandardized regression coefficient; LCI, lower bound of the 95% confidence interval (CI); UCI, upper bound of the 95% CI; GAD, generalized anxiety disorder. The main effects (not shown in this summary table) were adjusted for within each model testing a specific mediator.

Table S2

*Causal mediation analyses of pre-post trait mindfulness domains mediating the effect of MEMI vs. SM on pre-1MFU trait perseverative cognitions using non-linear generalized additive models*

|  | Predicting the pre-post mediator  (*a* path) | | |  | Predicting pre-1MFU  trait perseverative cognitions  (*b* path) | | |
| --- | --- | --- | --- | --- | --- | --- | --- |
|  | β | (LCI, UCI) | *p* |  | β | (LCI, UCI) | *p* |
| A. Observing | | | |  |  | | |
| Group x Time | 0.552^**^ | (0.201, 0.904) | .002 |  | -0.233^**^ | (-0.402, -0.064) | .007 |
| B. Describing | | | |  |  | | |
| Group x Time | 0.795 | (-0.491, 2.081) | .227 |  | -0.312^***^ | (-0.483, -0.141) | .000 |
| C. Acting with awareness | | | |  |  | | |
| Group x Time | 1.039 | (-0.276, 2.354) | .123 |  | -0.289^***^ | (-0.453, -0.126) | .001 |
| D. Non-judgment | | | |  |  | | |
| Group x Time | -0.404 | (-1.921, 1.114) | .602 |  | -0.375^***^ | (-0.533, -0.216) | .000 |
| E. Non-reactivity to inner experience | | | |  |  | | |
| Group x Time | 1.578^**^ | (0.529, 2.627) | .003 |  | -0.189^*^ | (-0.337, -0.041) | .013 |
| Spline regressions | | | |  | *F* | *p* |  |
| A. Observing | | | |  | 26.107^***^ | .000 |  |
| B. Describing | | | |  | 17.165^***^ | .000 |  |
| C. Acting with awareness | | | |  | 33.376^***^ | .000 |  |
| D. Non-judgment | | | |  | 90.501^***^ | .000 |  |
| E. Non-reactivity to inner experience | | | |  | 83.629^***^ | .000 |  |

*Note.* ^*^ *p* < .05; ^**^ *p* < .01; ^***^ *p* < .001.

MEMI, mindfulness ecological momentary intervention; SM, self-monitoring app; 1MFU, one-month follow-up; β, unstandardized regression coefficient; LCI, lower bound of the 95% confidence interval (CI); UCI, upper bound of the 95%. Trait perseverative cognitions was measured using the perseverative cognitions questionnaire. The main effects (not shown in this summary table) were adjusted for within each model testing a specific mediator.
